# Age exerts a continuous effect in the outcomes of Asian breast cancer patients treated with breast-conserving therapy

**DOI:** 10.1186/s40880-018-0310-3

**Published:** 2018-06-26

**Authors:** Fuh Yong Wong, Wei Ying Tham, Wen Long Nei, Cindy Lim, Hui Miao

**Affiliations:** 1 Division of Radiation Oncology, National Cancer Centre Singapore, 11 Hospital Drive, Singapore, 169610 Singapore; 2 Saw Swee Hock School of Public Health, National University of Singapore, Singapore, Singapore

**Keywords:** Breast cancer, Breast-conserving therapy, Locoregional recurrence, Breast cancer-specific survival, Breast cancer-free survival, Younger age

## Abstract

**Background:**

Asians are diagnosed with breast cancer at a younger age than Caucasians are. We studied the effect of age on locoregional recurrence and the survival of Asian breast cancer patients treated with breast-conserving therapy.

**Methods:**

Medical records of 2492 patients treated with breast-conserving therapy between 1989 and 2012 were reviewed. The Kaplan–Meier method was used to estimate locoregional recurrence, breast cancer-free survival, and breast cancer-specific survival rates. These rates were then compared using log-rank tests. Outcomes and age were modeled by Cox proportional hazards. Fractional polynomials were then used to test for non-linear relationships between age and outcomes.

**Results:**

Patients ≤ 40 years old were more likely to have locoregional recurrence than were older patients (Hazard ratio [HR] = 2.32, *P *< 0.001). Locoregional recurrence rates decreased year-on-year by 4% for patients with luminal-type breast cancers, compared with 8% for those with triple-negative cancers. Similarly, breast cancer-free survival rates increased year-on-year by 4% versus 8% for luminal-type and triple-negative cancers, respectively. Breast cancer-specific survival rates increased with age by 5% year-on-year. Both breast cancer-free survival and breast cancer-specific survival rates in patients with luminal cancers exhibited a non-linear (“L-shaped”) relationship—where decreasing age at presentation was associated with escalating risks of relapse and death. The influence of age on overall survival was confounded by competing non-cancer deaths in older women, resulting in a “U-shaped” relationship.

**Conclusions:**

Young Asian breast cancer patients have a continuous year-on-year increase in rates of disease relapse and cancer deaths compared with older patients with no apparent threshold.

## Background

Breast cancer is relatively uncommon in young women. According to the Surveillance, Epidemiology, and End Results (SEER) program database, only 6.5% of breast tumors occur in women age < 40 years and only 0.6% in women age < 30 years [1].

Young age is, however, an important independent poor prognostic factor for breast cancer. Several studies have shown that young breast cancer patients have poorer local disease control, increased breast cancer mortality, and reduced overall survival compared with older premenopausal or postmenopausal patients [2–9]. However, the definition of “young age” has been arbitrary, with various age cut-offs ranging from 30 to 40 years.

Young women with breast cancer are faced with a choice between mastectomy and breast-conserving therapy (BCT). Women who chose BCT reported better body image, sexual functioning, and fewer disruptions to lifestyles compared with those who underwent mastectomy [10]. In some studies, age has been shown to be a predictor for the choice of type of surgery [11]. Women who chose BCT were likely to be younger. Factors affecting women’s choice of surgery included the risk of local recurrence and fears about losing a breast [12].

Several studies have shown that breast cancer presents earlier in Asian women than in their Western counterparts [13, 14]. In addition, patients in developing countries who are diagnosed with breast cancer are approximately one decade younger than those in developed countries [15]. The proportion of young patients (< 35 years) varies from approximately 10% in developed countries to up to 25% in developing Asian countries [13–15]. In developing countries, the majority of breast cancer patients continue to be diagnosed at a relatively late stage, and locally advanced cancers constitute over 50% of all patients [13–15]. The clinicopathology profile of the young Asian breast cancer patient differs from that of patients elsewhere in the world [16].

In addition, young women form a socioeconomically important segment, in both developing and developed societies. They are economically productive and often have young families and developing careers. For young women, the knowledge that their youth predisposes them to a worse prognosis affects them in two ways: it affects them in a profound manner psychosocially and sexually, and it undoubtedly influences their choices regarding child bearing and future plans, which may affect their choice of treatment and compliance with treatment [17, 18].

Due to the large proportion of young breast cancer patients in our Asian population, with their preference for breast-conserving, we conducted this study to better understand the effect of age on Asian breast cancer patients and their outcomes after BCT [19].

## Patients and methods

### Patients and data collection

Retrospective chart reviews of breast cancer patients treated with BCT at the National Cancer Centre Singapore between 1989 and 2012 were performed. All patients treated with curative intent are included. Patients who received mastectomy up front or completion mastectomy for positive margins are excluded. BCT refers to the wide local excision of the tumor with appropriate management of axillary nodes, followed by adjuvant whole breast radiotherapy and systemic treatment when indicated. Patients were staged according to the 7th edition of the American Joint Committee on Cancer (AJCC) system.

### Follow-up and endpoints

Patients were followed up until death or until March 2013. Patients were seen at least twice a year in the first 5 years with digital breast examinations in the clinic. Mammograms and/or ultrasound of the breasts were scheduled annually in patients without symptoms. The endpoints studied were overall survival (OS), breast cancer-specific survival (BCSS), breast cancer-free interval (BCFI) and locoregional recurrence (LRR). Overall survival was defined as the time from diagnosis to death from any cause. BCSS was defined as the time from diagnosis to death from breast cancer-related events. BCFI referred to the time from surgery to the first breast cancer recurrence at any site. This included contralateral breast cancer and breast cancer-related deaths. LRR events comprised ipsilateral local, nodal, or locoregional recurrences. Concurrent local and distant recurrences were not considered local recurrences. Patients without events were censored at the time of their last follow-up.

### Statistical analyses

Patients were divided into two age groups—using 40 years as the cut-off point. Associations between patient characteristics and age group were tested using the Pearson’s Chi square test, the Fisher exact test, or the Wilcoxon rank sum test. The strength of association was estimated using the Cramer V test, the Kendall rank correlation coefficient, or the Spearman’s rank correlation coefficient. The variables analyzed included (1) tumor size; (2) the number of nodes involved; (3) histological subtypes (approximated from immunohistochemical assessment of hormone receptor and HER-2 status); and (4) tumor grade. The Kaplan–Meier method was used to determine survival estimates. The log-rank test was used to test the differences in survival between the two groups of patients. In addition, a patient’s age at diagnosis was analyzed as a continuous variable, using the Cox proportional hazards model. Fractional polynomials, with a maximum degree of 2, were used to test for non-linear relationships with age. The closed-test algorithm was used for fractional polynomial model selection [20]. A 2-sided *P* value of less than 0.05 was considered statistically significant. All analyses were performed in Stata 11.2 (StataCorp, College Station, Texas, USA).

### Relative survival analysis

A relative survival analysis was conducted to estimate the net survival and account for the variations in underlying background mortality of the different age groups. A 5-year relative survival ratio (RSR) was calculated as the ratio of observed 5-year cumulative survival of patients in the present study to the expected survival of the general population, matched by age and calendar year. The expected survival was derived from Singapore female population life tables, using the Ederer II method [21]. A generalized linear model for excess mortality was fitted, according to the Hakulinen and Tenkanen method, to compare excess mortality by age and provide estimates of excess hazard ratios, relative excess risk (RER). This fitting was performed with patients older than 60 years of age as the reference group while adjusting for follow-up time and histological subtype [22]. The data were then analyzed using Stata (Version 12.1; StataCorp).

## Results

### Patient demographics and treatment received

The study included 2492 patients who had BCT, 447 (17.9%) of whom were 40 years or younger at the age of diagnosis (Table 1). The median age at diagnosis was 49 years (22–92 years). The median follow-up period was 4.14 years (range 0.03–24.83). With the exception of 5 patients, all patients completed adjuvant radiotherapy; 96.7% (2410) of patients had axillary clearance or sentinel lymph node biopsy. All patients with positive sentinel lymph nodes received axillary clearance.

**Table 1:** Age distribution for the 2492 Asian patients with breast cancer

Age at diagnosis (years)	Number	%
≤ 30	66	2.7
31–40	381	15.3
41–50	953	38.2
51–60	738	29.6
61–70	293	11.8
71–80	56	2.3
> 80	5	0.2

The patients’ clinicopathologic characteristics are summarized in Table 2. Older patients were more often diagnosed with stage I disease (56.9% vs. 45.9%, *P *<0.001), while younger patients had a significantly higher proportion of Grade 3 (47.0% vs. 35.6%, *P *<0.001) and estrogen receptor (ER)-negative tumors (28.2% vs. 23.3%, *P* = 0.020). Younger patients were also more likely to have triple-negative breast cancers (15.2% vs. 9.9%, *P *<0.001) and lymphovascular invasion (22.6% vs. 18.4%; *P* = 0.012) than were older patients. More younger than older patients received chemotherapy (62.0% vs. 45.7%, *P *<0.001).

**Table 2:** Characteristics of the 2492 Asian breast cancer patients treated with breast-conserving therapy (1989–2012)

Characteristic	Age ≤ 40 years (*n* = 447)	Age > 40 years (*n* = 2045)	*P* value	Strength of association
Age (years)				
Median (range)	36 (22–40)	51 (41–92)		
Race [*n* (%)]				
Chinese	342 (76.5)	1673 (81.8)		
Indian	21 (4.7)	94 (4.6)		
Malay	40 (8.9)	184 (9.0)		
Others	44 (9.8)	94 (4.6)	< 0.001	0.089
T category [*n* (%)]				
T0/T1/T1a/T1b/T1mic/Tx	85 (19.0)	542 (26.5)		
T1c	203 (45.4)	912 (44.6)		
T2–T4	159 (36.6)	591 (28.9)	0.001	− 0.060
N category [*n* (%)]				
N0	311 (69.6)	1525 (74.6)		
N1/N1mic	100 (22.4)	391 (19.1)		
N2/N3	36 (8.1)	129 (6.3)	0.086	− 0.030
TNM stage [*n* (%)]				
Stage 1	205 (45.9)	1163 (56.9)		
Stage 2A/2B	204 (45.6)	739 (36.1)		
Stage 3A/3C	34 (7.6)	130 (6.4)	< 0.001	− 0.064
Unknown	4 (0.9)	13 (0.6)		
Size (cm)				
Median (range)	1.8 (0–7.5)	1.6 (0–8)	0.001	
Unknown	31 (7)	72 (3.5)		
Grade [*n* (%)]				
Grade 1	61 (13.6)	440 (21.5)		
Grade 2	149 (33.3)	769 (37.6)		
Grade 3	210 (47.0)	727 (35.6)	< 0.001	− 0.086
Unknown	27 (6.0)	109 (5.3)		
Extensive intraductal component [*n* (%)]				
Not present	326 (72.9)	1567 (76.6)		
Present	74 (16.6)	372 (18.2)	0.751	0.007
Not mentioned/missing	47 (10.5)	106 (5.3)		
Margins (mm)				
0–5	215 (48.1)	982 (48.0)		
6–10	112 (25.1)	533 (26.1)		
> 10	87 (19.5)	442 (21.6)	0.744	0.012
Unknown	33 (7.4)	88 (4.3)		
Lymphovascular invasion [*n* (%)]				
No	304 (68.0)	1562 (76.4)		
Yes	101 (22.6)	376 (18.4)	0.012	− 0.052
Unknown	42 (9.4)	107 (0.2)		
No. of positive nodes				
Median (range)	0 (0–28)	0 (0–40)	0.024	− 0.045
Unknown	0 (0)	4 (0.2)		
ER^a^ [*n* (%)]				
Negative	126 (28.2)	476 (23.3)		
Positive	284 (63.5)	1467 (71.7)	0.009	0.054
Equivocal	0 (0)	4 (0.2)		
Unknown	37 (8.3)	98 (4.8)		
PR^a^ [*n* (%)]				
Negative	141 (31.5)	605 (29.6)		
Positive	267 (59.7)	1332 (65.1)	0.190	0.027
Equivocal	0 (0)	4 (0.2)		
Unknown	39 (8.7)	104 (4.8)		
HER2^a^ [*n* (%)]				
Negative	274 (61.3)	1365 (66.7)		
Positive	82 (18.3)	351 (17.2)	0.276	− 0.024
Equivocal	17 (3.8)	91 (4.4)		
Unknown	74 (16.6)	238 (11.6)		
Histologic subtype [*n* (%)]				
Luminal A/B	266 (59.5)	1421 (69.5)		
HER2 enriched	34 (7.6)	129 (6.3)		
Basal	68 (15.2)	202 (9.9)	< 0.001	0.087
Unknown	79 (17.7)	293 (14.3)		
Chemotherapy [*n* (%)]				
No	168 (37.6)	1106 (54.1)		
Yes	277 (62.0)	934 (45.7)	< 0.001	− 0.126
Unknown	2 (0.4)	5 (0.2)		
First event [*n* (%)]				
Locoregional recurrence	30 (6.7)	56 (2.7)		
Distant recurrence	38 (8.5)	91 (4.5)		
New contralateral breast cancer	17 (3.8)	43 (2.1)		
New non-breast or unknown cancers	5 (1.1)	40 (2.0)		
Death (any cause)	2 (0.5)	23 (1.1)		
Alive without disease	355 (79.4)	1792 (87.6)	< 0.001	0.125
Follow-up time (years)				
Median (range)	4.36 (0.18–24.83)	4.09 (0.03–22.14)	0.072	− 0.036

Patients were staged according to the 7th edition of the American Joint Committee on Cancer (AJCC) system

*ER* estrogen receptor, *PR* progesterone receptor

^a^Equivocal was excluded from the test of association

### Overall survival

**Fig. 1:**
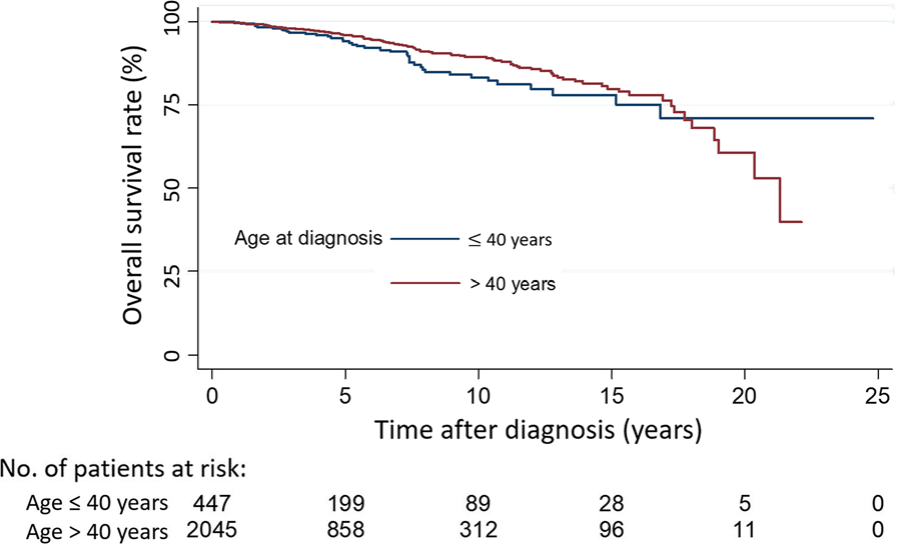
Kaplan-Meier overall survival estimates of patients ≤ 40 years old vs. those of patients > 40 years old. The hazard ratio of overall survival in the group older than 40 was 1.27 (P = 0.198) compared to the younger age group. 95% CI 95% confidence interval. Luminal: estrogen receptor (ER) or progesterone receptor (ER) positive breast cancers, HER2: HER2 amplified or over-expressed breast cancers and Basal: Triple negative breast cancers

**Fig. 2:**
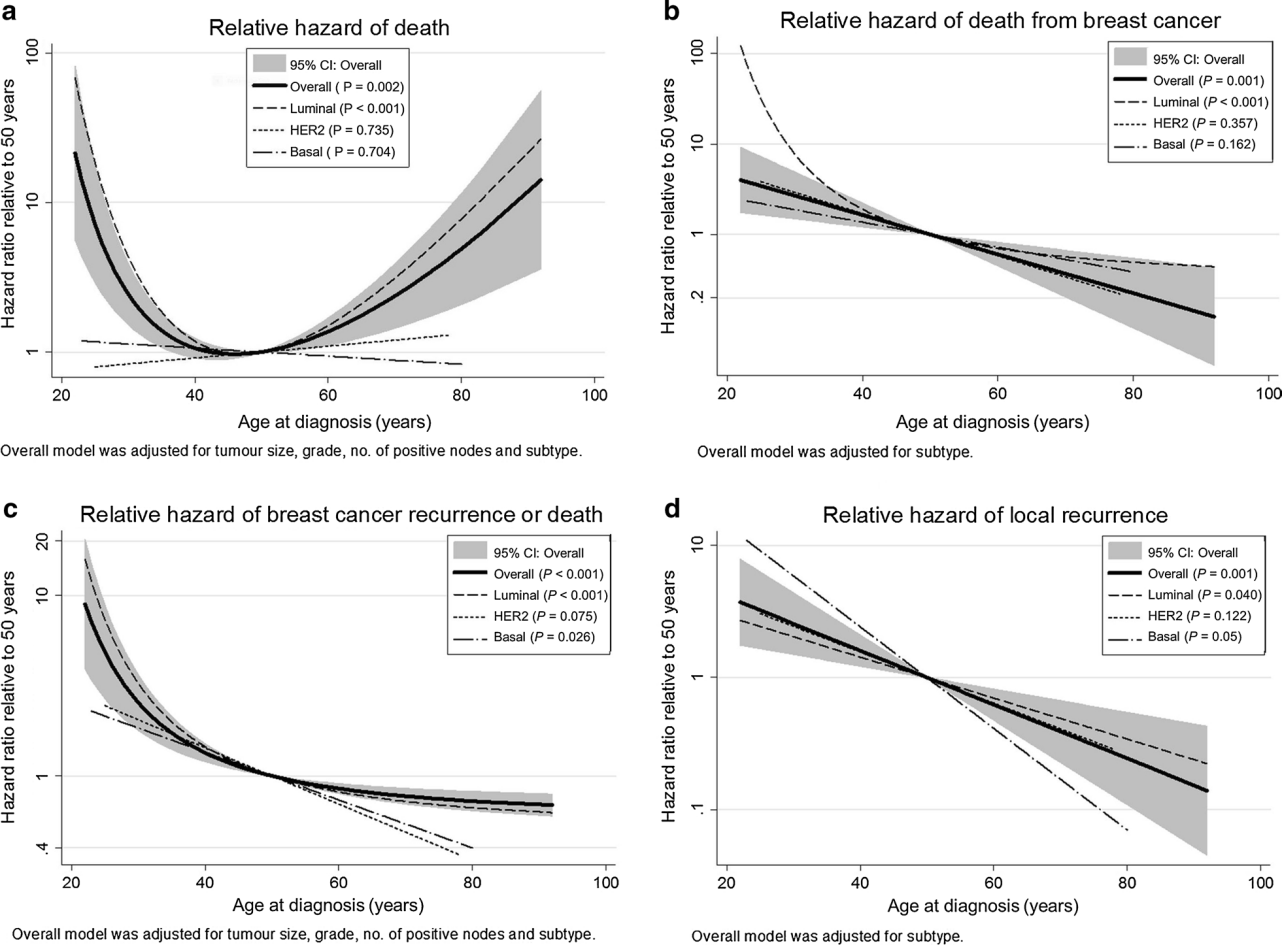
Plot for age at diagnosis and hazard ratio of over survival (**a**), breast cancer-specific survival (**b**), breast cancer-free interval (**c**), and breast cancer-free interval (**d**) with the baseline set at 50. Overall: Plot of hazard ratio of any deaths against age at diagnosis with the baseline set at age 50 for all histologic subtypes and 95% confidence interval (CI) shaded in gray. Separate plots for each histologic subtype, including luminal: estrogen receptor (ER) or progesterone receptor (ER) positive breast cancers; HER2: HER2 amplified or over-expressed breast cancers; and Basal: Triple-negative breast cancers superimposed

The OS rates were similar for older and younger patients; the HR of OS for older patients was slightly higher but not significant statistically (HR = 1.27 *P* = 0.198; Table 3, Fig. 1). However, when age was modeled as a continuous variable, we found that the relationship was significantly non-linear. A plot of the best fit fractional polynomial showed a U-shaped relationship between log relative hazard and age, indicating that the hazard of death first decreased and then increased with age, with the minimum at approximately 45 years (Fig. 2a). This trend may explain why age was not significant when modeled linearly or as 2 groups. The finding suggests that after age 45, there are increasingly larger competing background risks of death that exert a stronger influence on overall survival than on breast cancer deaths alone. When analyzed according to subtype, the luminal subtypes also showed a U-shaped relationship. HER2 and basal subtypes did not reach significance (Table 4).

**Table 3:** Five-year survival/failure rates and hazard ratios by age group for the 2492 Asian breast cancer patients treated with breast-conserving therapy (1989–2012)

Clinical outcomes	No. of events/no. of patients	5-year rate [% (95% CI)]	Log-rank, *P* value	Hazard ratio (95% CI)	Cox model *P* value
Overall survival					
All patients	162/2492	95.7 (94.6–96.6)			
≤ 40 years at diagnosis	40/447	94.1 (90.6–96.4)		1.27 (0.88–1.82)	
> 40 years at diagnosis	122/2045	96.1 (94.9–97.0)	0.198	1.00	0.209
Breast cancer-specific survival					
All patients	77/2492	98.0 (97.1–98.6)			
≤ 40 years at diagnosis	26/447	96.7 (93.7–98.3)		2.00 (1.23–3.23)	
> 40 years at diagnosis	51/2045	98.3 (97.4–98.9)	0.004	1.00	0.007
Breast cancer-free interval					
All patients	277/2492	10.5 (9.0–12.1)			
≤ 40 years at diagnosis	85/447	15.3 (11.6–20.1)		1.92 (1.49–2.50)	
> 40 years at diagnosis	192/2045	9.4 (7.9–11.1)	< 0.001	1.00	< 0.001
Local recurrence					
All patients	89/2492	3.4 (2.6–4.4)			
≤ 40 years at diagnosis	31/447	5.2 (3.1–8.5)		2.33 (1.49–3.57)	
> 40 years at diagnosis	58/2045	3.0 (2.2–4.1)	< 0.001	1.00	< 0.001

Proportional hazards assumption was violated for overall survival

**Table 4:** Best fit multivariate models for the 2492 Asian breast cancer patients treated with breast-conserving therapy (1989–2012)

Clinicopathologic characteristics	Overall survival		Breast cancer-specific survival		Breast cancer-free interval		Time to local recurrence	
	Hazard ratio (95% CI)	*P* value	Hazard ratio (95% CI)	*P* value	Hazard ratio (95% CI)	*P* value	Hazard ratio (95% CI)	*P* value
No. of events/no. of patients	98/1988		54/2120		182/1988		64/2120	
Age at diagnosis	25.21 [(age/10)^−2 ^− 0.04] + 0.06 [(age/10)^2 ^− 25]^a^	< 0.001	0.95 (0.92–0.98)	0.001	13.14 [(age/10)^−2 ^− 0.04]^a^	< 0.001	0.95 (0.93–0.98)	< 0.001
Tumor size	1.21 (0.99–1.46)	0.064	Not included		1.15 (1.00–1.32)	0.050	Not included	
Grade		< 0.001	Not included			< 0.001	Not included	
1	1				1			
2	4.06 (1.59–10.38)				3.38 (1.78–6.41)			
3	4.94 (1.90–12.84)				4.08 (2.12–7.86)			
No. of positive nodes	1.05 (1.01–1.09)	0.031	Not included		1.06 (1.03–1.09)	0.001	Not included	
Histologic subtype		0.024		0.002		0.007		0.002
Luminal A/B	1		1		1		1	
HER2 enriched	1.02 (0.50–2.12)		0.80 (0.24–2.64)		1.39 (0.85–2.28)		3.42 (1.82–6.41)	
Basal	1.99 (1.22–3.25)		2.94 (1.64–5.28)		1.85 (1.27–2.69)		1.61 (0.82–3.15)	

^a^Where age was significantly non-linear, the fractional polynomial function was presented instead of the hazard ratio. Age was scaled by a factor of 10 and centered on 50 years in these models. Where the number of events was insufficient to include all the covariates in the model, only age and histologic subtype were included

### Relative survival ratio

The 5-year relative survival ratio increased with age (Table 5). After adjusting for histological subtype and follow-up time, women younger than 45 years had the highest excess mortality (Relative Excess Risk 1.85, 95% CI 0.45–7.63), while the least excess mortality was observed for women aged between 51 and 60 years (RER 0.39, 95% CI 0.05–2.88). However, the differences were not significant in any age group.

**Table 5:** Relative survival ratio by age for the 2492 Asian breast cancer patients treated with breast-conserving therapy (1989–2012)

Age group	5-year cumulative survival rate (%)		5-year RSR [%(95% CI)]
	Observed	Expected	
≤ 40	94.3	99.7	94.6 (91.2–96.8)
41–50	96.7	99.2	97.4 (95.7–98.6)
51–60	96.9	98.1	98.8 (96.6–100.1)
> 60	92.3	93.0	99.2 (94.1–102.5)

*RSR* relative survival ratio

### Breast cancer-specific survival

Overall, 77 patients died from breast cancer-related events. Twenty-six of these patients were aged 40 or younger when diagnosed with breast cancer, while 51 were older than 40 years. Patients in the younger age group were more likely to die from a breast cancer- related event compared with those diagnosed later (HR = 2.0; 95% CI = 1.23–3.23; *P* = 0.004). Five-year BCSS rates were 96.7% and 98.3% (*P* = 0.004) for patients age ≤ 40 and > 40 years, respectively (Table 3).

When age was analyzed as a continuous variable, there was no evidence of a non-linear relationship between BCSS and age. The linear model was chosen by the model selection algorithm; it was estimated that the hazard of BCSS decreased linearly with increasing age by approximately 5% per year (95% CI = 2%–8%; *P* = 0.001). Therefore, the younger patient has a higher risk of dying from breast cancer compared to an otherwise similar but older patient; those patients at the youngest of age would have the highest risk of breast cancer death amongst all. Luminal breast cancers had a significant non-linear relationship with the hazard decreasing rapidly up until approximately age 50 at diagnosis and then more gradually thereafter. HER2 and triple-negative subtypes did not reach significance (Table 4, Fig. 2b).

### Breast cancer-free interval

A total of 277 patients developed breast cancer-related events. Of these patients, 85 were 40 years or younger at diagnosis, while the remaining 192 were older than 40 years. Patients 40 years or younger at diagnosis were more likely to have breast cancer recurrence compared with patients who were older at diagnosis (HR = 1.92; CI 1.49–2.50; *P *<0.001; Table 3).

When age was analyzed as a continuous variable, the relationship between BCFI and age was significantly non-linear. A plot of the best-fit fractional polynomial showed an L-shaped relationship between log relative hazard and age, indicating that an older age at diagnosis was associated with a lower hazard of breast cancer recurrence or death. Following an initial rapid decrease in the hazard with increasing age at presentation, the curve become more gradual at approximately 40 years of age, which suggests that beyond this point, the influence of age on breast cancer recurrence or death is diminished.

When we analyzed the histological subtypes, all three—Luminal A/B, HER2 enriched, and Basal subtypes—demonstrated increased risk in younger patients. A similar L-shaped relationship was found in luminal cancers, but non-linear relationships were not detected in HER2 and triple-negative subtypes. The hazard ratio for the basal subtype was 0.92, while the hazard ratio for the luminal subtype was 0.96. The decrease in the hazard each year related to the increase in age at diagnosis was larger for the basal subtype (8% decrease/year), than for the luminal subtype (4% decrease/year). The hazard decreased faster with age at diagnosis for the basal subtype than for the luminal subtype (Table 4 and Fig. 2c).

### Local recurrence

Eighty-nine patients had local recurrences. Thirty-one of these patients were 40 years old or younger, while 58 of them were older than 40. The 5-year LRR rates were 5.2% and 3.0% (*P *<0.001), respectively, for the ≤ 40 and > 40 age groups. Patients ≤ 40 years old were approximately twice as likely as their older counterparts to have a local disease recurrence (HR = 2.33, P < 0.001).

There was no evidence of non-linearity when age was analyzed as a continuous variable. The hazard of LRR decreased linearly with increasing age (*P* = 0.001), from a 4% decrease/year (95% CI = 1–7%, *P* = 0.040) in the luminal subtype to an 8% decrease/year (95% CI = 2–14%, *P* = 0.005) in the triple-negative subtype. A multivariate analysis showed that age remained a significant factor in patients for local recurrence after controlling for histological subtype (Table 4, Fig. 2d).

## Discussion

Our study shows that young Asian breast cancer patients treated with BCT have higher rates of local recurrence and breast cancer death than other patients. While previous investigations have examined the effect of age in a dichotomous fashion using arbitrary definitions of youth, our results showed no apparent threshold effect of age on breast cancer control or survival.

Outcomes of patients with breast cancer are influenced by the complex interactions between tumor biology, host biology and treatment received. Many aspects of tumor biology that influence treatment responses and outcomes have been clearly established, including (1) the stage of disease at presentation, (2) tumor grade, (3) the presence of hormone receptors, and (4) HER2 overexpression. Although many of these factors are associated with a patient’s age and account for a significant portion of the variability in outcomes, age still remains a significant, independent prognostic factor [23].

The actual mechanism through which age influences outcomes is unknown. Recent studies have shown that breast cancer in young patients is replete with processes related to immature mammary epithelial cells (luminal progenitors, mammary stem, c-kit, and Receptor activator of nuclear factor kappa-Β ligand RANKL), growth factor signaling and mitogen activated protein kinase (MAPK), and phosphoinositide 3-kinase (PI3K)-related pathways [24–28]. Other studies that contradict the abovementioned reasoning postulate that age is no longer a significant prognostic factor, after correcting for clinicopathological and histopathological features such as grade, nodal status, ER status, and breast cancer subtypes [28, 29]. However, this argument only brings us back to the question of why younger women are more prone to aggressive subtypes of breast cancer in the first place and how the factors associated with younger patients, such as increased breast density and lower parity, contribute to these findings.

Although the same general trend for the age-outcome relationship is observed, it is clear that this interaction is complex and may well involve different mechanisms for each of the well-recognized breast cancer subtypes. The relationships of disease control and cancer death to age in luminal cancers are L-shaped, emphasizing the preponderance of risks in the youngest patients. This inflection point at 40–45 years of age may be indicative of a switch in factors driving disease initiation and progression. It has been similarly observed that luminal B cancers are particularly associated with poor outcomes in young breast cancer patients [30, 31]. However, the relationship between age and breast cancer events in HER2 enriched and triple-negative subtypes are more manifestly linear.

Our findings build on earlier studies carried out in dissimilar populations, indicating that women diagnosed with breast cancer at a younger age are more likely to have a poorer outcome. In patients younger than 40 years old, adjuvant radiotherapy following breast-conserving surgery reduced this risk by more than half [29, 30].

One study of 1703 patients from a single center showed that the relationship between recurrence hazard and age was continuous. Fitting with a log-linear function showed a 4% decrease in recurrence and a 2% decrease in cancer-specific death for every one-year increase in age, thus echoing our study findings [6].

A larger study carried out in Korea by Han et al. [7] showed that in patients younger than 35 years of age, there was an increasing risk of death with decreasing age; however, age did not affect patients between 35 and 50 years old. This finding is similar to our results for patients in the same age range. However, Han et al. [7] only included patients up to 50 years of age, whereas our study examined a wider age range. Our study examined records of patients older than 80 and showed that patients diagnosed at ages older than 50 faced increasing competing risks of death from non-breast cancer mortality; the evidence was a larger difference between breast cancer-specific survival and overall mortality in the older age group.

A relative survival analysis in our study population yielded results different from those reported in previous studies. Younger patients were found to have increased excess mortality compared with the older age groups, although this finding was not statistically significant in our study. The lack of statistical significance was possibly due to the smaller number of patients in the younger age group compared with that in the older age group, as well as the small number of events in the older age group.

Chia et al. [34] studied Singaporean breast cancer data and performed a population-based survival analysis. This study showed that younger patients have higher relative survival rates and lower excess risks of death compared with older patients. As demonstrated, this conclusion is opposite to that reached by our group. One possible explanation could be that the study by Chia et al. was conducted over an earlier period (1968–1992) that observed less effective systemic therapy for older patients. Older patients were often undertreated due to poorer health, reduced acceptance of treatment, or the denial of standard treatment arising from concerns of poorer tolerance to toxicity. In contrast, the increased use of systemic treatment, more effective chemotherapy, and better supportive care among older patients during our study period (1989–2012) enables older patients to enjoy the benefits of more effective treatment and improved outcomes. This finding reflects the relatively more indolent nature of their disease. A smaller, single-institution study by Foo et al. [35] showed that patients younger than 40 years did not have poorer overall survival, despite having tumors and a poorer prognostic profile. This result may be attributed to the more aggressive treatment the patients received. It is therefore likely that the differences in outcomes between age groups can be diminished with better treatment and better cancer subtyping.

These studies raise the issue of how we should define the relationship between age and the management of patients with breast cancer. Younger patients may need more aggressive treatment, while older patients may need less aggressive treatment. The current literature reveals conflicting results with regard to locoregional control in patients who have received BCT. Some studies have observed an increased risk of local recurrence with BCT, while others have not [32–36]. Nonetheless, there is no evidence that survival rates are inferior for younger patients who have received BCT relative to those for patients undergoing mastectomy [33]. Therefore, young age is not a contraindication to BCT.

Understanding the effect of age on breast cancer may allow us to better select patients for more appropriate therapy. Regan et al. studied SOFT and TEXT trials and found that the clinicopathologic characteristics that had the greatest contribution to the composite measure of recurrence risk relative to the complementary reference categories were young age (less than 35 years), four or more positive lymph nodes, and Grade 2–3 tumors. There was a gradual reduction in the hazard ratio from 2.2 to 1.2 for women 35 or younger to older than 50. It is now recommended that young women younger than 35 with hormone receptor positive breast cancer receive tamoxifen or exemestane plus ovarian suppression [37, 38].

At the other end of the spectrum, studies have shown that the survival of patients > 70 years old with estrogen-receptor (ER) positive tumors was not improved by the addition of adjuvant radiotherapy on top of lumpectomy and hormonal therapy; such an approach may therefore represent overtreatment in women of this age group [39, 40].

Mao et al. [41] showed that for women diagnosed at age 60 or younger, only the luminal A and basal molecular subtypes showed an overall survival benefit from radiotherapy. For women diagnosed at age 60 and older, there was no significant overall survival benefit of radiotherapy across all molecular subtypes.

Our study examined breast cancer outcomes with BCT in the Asian population. As the median age of diagnosis of breast cancer in Asians is 10 years younger than that in the Caucasian population, it is important for us to understand the effects of age on breast cancer. However, a drawback of our study was that the median follow-up period was only 4 years. A longer follow-up study in this population is being planned and would give us more information on the long-term outcome of BCT.

By intentionally limiting our study patients to only those with BCT, we may have inadvertently underestimated the effect of age, as long-term observational studies have shown improved overall outcomes with BCT compared with mastectomy. van Maaren et al. [42] carried out a population-based study on the Netherlands Cancer Registry and found that the 10-year overall survival for patients who received BCT was 21% higher than that for those who received mastectomy. The results were similar across all T and N stages. In particular, patients with T1N0 breast cancers had a 24% higher metastasis-free survival after BCT compared with that for patients undergoing mastectomy. In addition to our shorter follow-up, this observation may further explain the relatively high BCSS for patients in our study cohort, among whom more than half had Stage I cancer.

Our study population has a relatively small number of patients with HER2 or basal subtypes breast cancers. As such, the relationship between age and clinical outcomes cannot be determined accurately. To a large extent, our results have been affected by the large proportion of patients with luminal cancers. In the model construction, there might have been non-linear relationships that we missed, as we used significance testing (which is sensitive to sample size) to select models.

There were sufficient events to perform multivariate analysis for the relationship between age, overall survival and breast cancer-free interval. For other endpoints, these variables could not be adjusted for as the number of events was too small, particularly as size and number of positive nodes were modeled as continuous variables.

## Conclusions

In conclusion, our study shows that young Asian breast cancer patients treated with BCT have higher local recurrence rates and rates of breast cancer death than do older patients. This effect is shown to be continuous, where every 1-year increase in age at presentation increases local control and decreases breast cancer death. The subtypes of breast cancers may display differing age-outcome relationships, reflecting differences in neoplastic mechanisms.

## Abbreviations

BCT: breast-conserving therapy
HR: hazard ratio
ER: estrogen receptor
SEER: surveillance, epidemiology and end results
BCFS: breast-cancer-free-survival
LRR: locoregional recurrence
RSR: relative survival ratio
BCSS: breast cancer-specific survival
MAPK: mitogen activated protein kinase
RANKL: receptor activator of nuclear factor kappa-Β ligand
